# Long-Term Impact of Global Pediatrics Curriculum, Experience, and Mentorship in Pediatric Residency

**DOI:** 10.4269/ajtmh.21-1014

**Published:** 2022-02-07

**Authors:** Ifelayo Ojo, Andrew Wu, Stephanie Lauden, Tina Slusher, Sophia Gladding, Emily Danich, Cynthia Howard

**Affiliations:** ^1^ University of Minnesota, Department of Pediatrics, Minneapolis, Minnesota;; ^2^ Hennepin Healthcare, Department of Pediatrics, Minneapolis, Minnesota;; ^3^ Boston Children’s Hospital, Boston, Massachusetts;; ^4^ Nationwide Children’s Hospital, The Ohio State University, Columbus, Ohio;; ^5^ University of Minnesota, Department of Medicine, Minneapolis, Minnesota

## Abstract

Global health education is offered increasingly during residency training. The University of Minnesota has offered a global pediatrics track to residents since 2005. This study aimed to understand the impacts of a global pediatrics track on graduates’ career choices, skills, and current engagement in global health. An electronic survey was sent to 110 track graduates in February to April 2020. Data were analyzed with descriptive statistics and paired *t*-tests. Content analysis of written comments was conducted. The response rate was 62% overall, varying by question. Overall, 75% of responding graduates reported global pediatrics track participation affected their career choices. Eighty-four percent recalled plans to work in global health after graduation and 64% of respondents reported working in global health abroad or at home at the time of the survey. Incorporation of public health and global research represented the greatest percentage change in career plans from the time of enrollment to graduation (24% and 27%, respectively). Ninety-five percent of respondents reported that track participation improved their ability to elicit information about cultural beliefs and practices, and 86% reported improvement in cost-conscious care. An increase in global health knowledge and skills was the most common category of impact cited by respondents. Neonatal resuscitation, bubble continuous positive airway pressure, and homemade spacers for metered-dose inhalers were the most used global health-adapted skills. Our study found that graduates of the global pediatrics track perceive their participation affected their knowledge, skills, and attitudes positively, with the potential to improve clinical care and promote health equity locally and globally.

## INTRODUCTION

Global child health education has been defined as “the study of diseases and their social determinants (which affect most children worldwide and are not contained by borders), the development of cultural humility, and the acquisition of a skill set for work in resource-limited settings.” ^1^ Many American pediatric residency programs combine the curricula, skills training, experiences, and mentorship required to achieve the goals of global child health education into tracks or certificate programs. ^2^ Approximately one quarter of all pediatric residency programs in the United States reported having a dedicated global pediatrics track in a 2014 survey. ^3^

The University of Minnesota has offered a global pediatrics track since 2005. It is an opt-in residency track offered to all pediatric and medicine–pediatric residents. There is no cap on enrollment. On average, 53% of pediatric residents and 65% of medicine–pediatric residents participate in the track every year. To earn a global pediatrics track certificate at the University of Minnesota, participants are expected 1) to participate in an educational curriculum including a core set of required sessions; 2) to complete pre-travel preparation, which includes skills training as well as mental and emotional preparedness through simulations; 3) to complete successfully a global child health elective and associated assignments, including an academic project and engagement in post-elective debriefing; and 4) to present their academic project at pediatric grand rounds or at a poster session during the global pediatrics track graduation. Further curriculum details are found in The Minnesota Model. ^4^

The first cohort of pediatric and medicine–pediatric residents graduated in 2008. Since then, an average of 54% of residents on the track completed the requirements to receive a global pediatrics track certificate. The skills training component of the pre-travel preparation evolved to become a stand-alone curriculum known as the Procedural Education for Adaptation to Resource-Limited Settings (PEARLS). ^5^ PEARLS is a modular package of procedural skills taught in a hands-on workshop during which participants are given the opportunity to practice skills prior to their global pediatric elective. ^6^

As opportunities in global pediatrics training have expanded, there have been increasing calls for more robust evaluation of the outcomes of such training. Understanding outcomes of global health training can inform further development of global pediatrics education. ^1^^,^ ^7^ To date, evaluations of global health education have focused mainly on immediate outcomes, such as changes in medical knowledge, clinical skills, and career plans. Most of these outcomes have been evaluated shortly after trainees participate in a global health elective or training program, with few studies evaluating longer term impacts of global health participation. ^8^^–^ ^10^ The impacts of global health residency tracks on post-residency career choices, long-term engagement in global health, and changes in clinical practice have not been widely studied.

The purpose of our study was two-fold. First, we sought to determine the perceived impact of the University of Minnesota’s global pediatrics track on career plans, practice of medicine, and skills of graduates who received a certificate in global pediatrics. Second, we sought to understand and quantify more fully the procedures and skills graduates have used in resource-constrained settings to inform further development of the PEARLS curriculum and to identify which procedures should be prioritized in future pre-departure training.

## MATERIALS AND METHODS

### Setting and sample.

On the 15th anniversary of the integration of the global pediatrics track curriculum into the pediatric residency program at the University of Minnesota, we initiated our effort to assess the impact of the curriculum on track graduates. An e-mail was sent to all individuals who received a certificate in global pediatrics between 2008 and 2019 for whom we had a valid e-mail address. As of June 2019, 121 physicians from 11 residency cohorts had completed the track requirements successfully and received a certificate in global pediatrics upon graduation from residency. Of the 121 graduates, the survey was successfully sent electronically to 110 graduates. We sent up to three reminders between February and April 2020. Participation in the survey was voluntary and anonymous.

### Survey development.

All questions were created and developed by the authors, who include faculty with expertise in global health curriculum development, curriculum evaluation, and qualitative research methodology. Three of the authors (I. O., A. W., and S. L.) are track graduates. The questions were informed by previous studies of outcomes of global health education. ^11^^,^ ^12^ The 13 questions were designed to collect data on the track graduates’ perceptions regarding the impact of their global pediatrics training on their career choices, current involvement in global health work, clinical skills, and use of the track’s procedural skills curriculum (PEARLS) while abroad. Survey questions were pilot-tested by six track graduates. Based on their feedback, the questions were refined and finalized (Supplemental Appendix 1).

The survey was created using Qualtrics (Qualtrics, Provo, UT), ^13^ a web-based tool for creating surveys and the standard survey software at the University of Minnesota. Each participant received an e-mail through Qualtrics with a template message from the study team and a hyperlink to take the survey in Qualtrics.

The University of Minnesota institutional review board reviewed the study and determined the activity did not meet criteria for human subjects’ research and was therefore exempt from review board approval.

### Data analysis.

We calculated descriptive statistics for the responses to each survey question and compared the retrospective pre- and post-track participation questions using paired *t*-tests. All statistical analyses were conducted using Microsoft Excel 365. To analyze written responses to the open-ended questions “Describe any other impacts of global pediatrics training” and “Please provide more information about any of the skills above or comment on how the global pediatrics training has influenced your practice of medicine,” we conducted qualitative content analysis. ^14^ Three authors with qualitative research experience (C.H., S.L., and S.G.) read and coded the written responses and clustered their codes independently into initial categories that described the areas of impact identified in the written responses. They then met and compared their initial codes and categories until agreement was reached on a shared set of categories. The authors then re-read and coded independently the written responses to the categories contained in the shared set of categories. After coding these responses independently, the authors compared their coding of the responses to the categories and discussed any disagreements in coding until consensus was reached. The frequency of each category was calculated (i.e., the number of comments that contained each category). The qualitative analysis was conducted using Microsoft Word.

## RESULTS

Sixty-eight track graduates completed the survey (response rate, 62%). Responses to individual questions were not mandatory, so response rates varied per question, as indicated in the tables for each question or set of questions.

### Impact on career plans.

Overall, 51 respondents (75%) reported that participation in the global pediatrics track affected their long-term career choice. Of the 45 respondents to the questions about career plans before and after participating in the track, the majority (84%) indicated they planned to work in global health both before and after their global pediatrics track experience. Although the overall percentage was unchanged, six of the respondents did change their career plans, with three deciding to work in global health after track participation. A majority also reported plans to work with underserved populations globally and domestically, which increased after participation in the track, although the changes were not statistically significant. Although a relatively low number of respondents reported plans to incorporate public health or research into their careers before participating in the track, many respondents reported increased interest in public health and research after track participation (*P* > 0.05). Almost half of the respondents reported plans to pursue sub-specialty training before participating in the track, which decreased, not significantly, after participation in the track (Table 1).

**Table 1:** Career plans of global pediatrics track graduates before and after participation (45 respondents)

Career plans	Pre-track, n (%)	Post-track, n (%)	*P* value
Global health work	38 (84)	38 (84)	1
Global underserved populations	38 (84)	40 (89)	0.78
Domestic underserved populations	36 (80)	41 (91)	0.48
Incorporation of public health into clinical work	25 (56)	36 (80)	0.13
Inclusion of research as part of career	11 (24)	21 (47)	0.1
Subspecialty training	18 (40)	17 (38)	0.87

### Current involvement in global health work.

**Figure 1.**
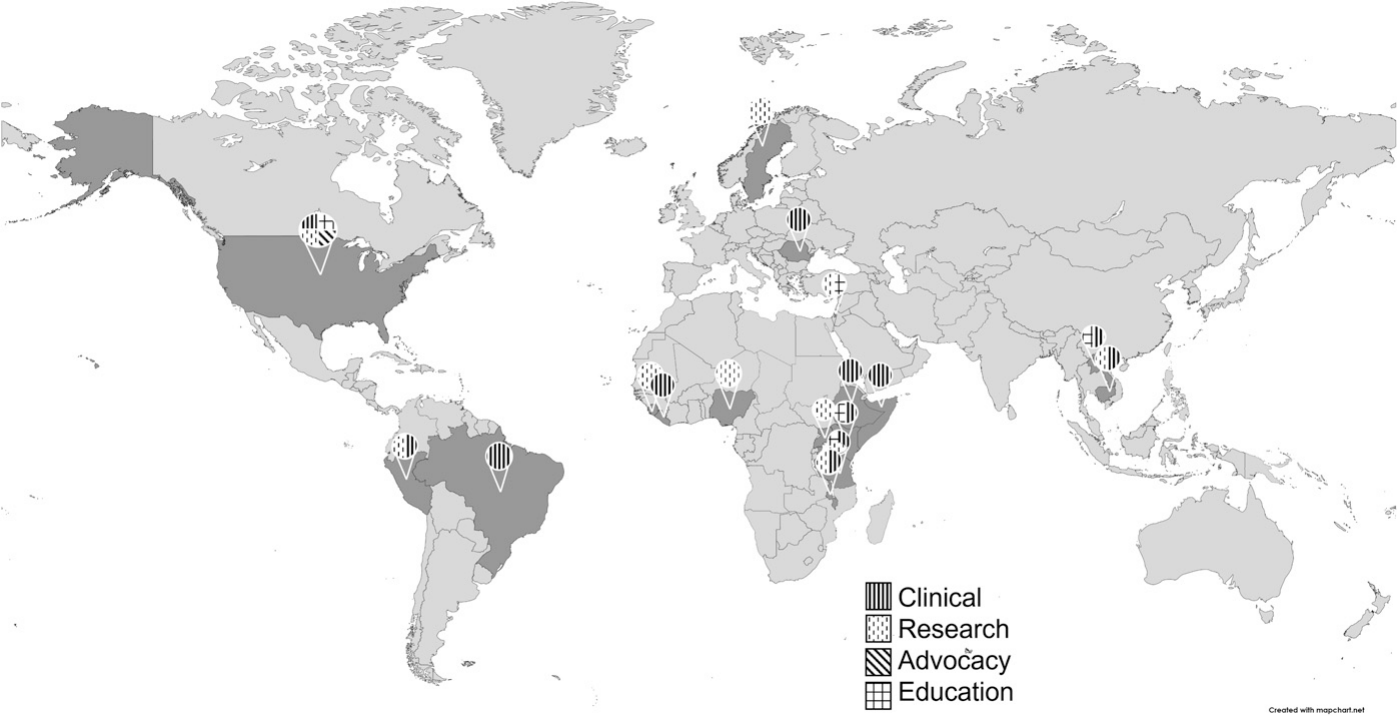
Geographic representation and type of global health work performed by respondents. Respondents were working in 17 countries at the time of completing the survey. The countries highlighted in the figure are Brazil, Cambodia, Ethiopia, Kenya, Laos, Liberia, Malawi, Nigeria, Palestine, Peru, Romania, Sierra Leone, Somaliland, Sweden, Tanzania, Uganda, and the United States. Type of work performed varied from country to country and included advocacy, clinical, education, and research. The size and division of circles in the figure do not represent the proportion of global health work performed in the countries.

Of 58 respondents, 64% (37 of 58) reported they were currently engaged in global health-related work. Respondents were working in 17 countries at the time they completed the survey. Respondents reported being engaged in different areas of global health, including clinical practice, education, research, leadership, and advocacy, with some respondents reporting being involved in two or more areas. The work in which respondents are engaged within each of these areas is described in more detail later. Figure 1 presents the geographic representation and type of global health work performed by respondents.

#### Clinical practice.

Of the 37 respondents, 59% reported their global health work as being either partially or entirely clinical in the United States or abroad. In the United States, the following clinical work settings were identified: federally qualified health center and primary care practice servicing a high number of immigrant patients, as well as the National Health Service Corps in California with immigrants primarily from Central and South America. Internationally, respondents reported clinical work with a non-governmental organization in Liberia; as well as remote clinical work by a pediatric cardiologist, who reads echocardiograms for a hospital in Kenya where they had previously worked and taught in person. In the United States, 10 respondents (27%) were working within immigrant or Native American communities. In addition to the cardiologist, a rheumatologist, neonatologist, and infectious disease specialist reported being actively involved in global health work.

#### Education.

Twenty-seven percent of respondents reported educating medical students and residents in global child health. Noted were educational activities in the United States and three locations abroad: the Middle East, Africa, and Southeast Asia. Clinical education in pediatric medicine and, specifically, in emergency medicine were reported. These opportunities were through academic institutions, non-governmental organizations, and one academic institutional partnership with a ministry of health. Domestic global health education was implemented primarily in residency programs.

#### Research.

Thirty percent of respondents were involved in global health research. Research abroad included work in Tanzania with NIH funding, research in Uganda and Cambodia, the study of malaria and malaria vaccines in Malawi, and research with non-governmental organizations doing international development work (country not specified).

#### Leadership.

Leadership positions that were reported included university faculty appointments in global health education, board membership for a not-for-profit global health organization, leadership positions in clinics serving low-income families, and leadership in global health research.

#### Advocacy.

One respondent reported advocacy work for immigrant rights. Another respondent reported health-care capacity building in pediatric medicine and public health in Palestine.

Three respondents reported current work in global health but did not identify specific activities or location.

### Impact on clinical skills.

Of the 58 respondents to questions about the relationship between global pediatrics track participation and clinical skill development, the majority reported that participating in the track improved their skills in eliciting information about cultural beliefs and practices (95%), cost-conscious care (86%), physical examination skills (79%), understanding medical terms in a non-English language (72%), and recognizing sick patients (those requiring acute or immediate medical attention) versus non-sick patients (those appearing physiologically stable) (62%) (Table 2).

**Table 2:** Clinical skills attributed to global pediatrics track participation (58 respondents)

Did the global pediatrics track improve your skills in the following?	Yes, n (%)	No, n (%)	Unsure, n (%)
Eliciting information about cultural beliefs and practices	55 (95)	2 (3)	1 (2)
Cost-conscious care	50 (86)	6 (10)	2 (3)
Physical examination	46 (79)	5 (9)	7 (12)
Understanding medical terms in a non-English language	41 (71)	13 (22)	4 (7)
Recognizing sick vs. non-sick patients	36 (62)	15 (26)	7 (12)

### Additional impacts of global pediatrics training.

Through qualitative analysis of 29 written responses to the two open-ended questions asking respondents to describe additional impacts or influences on their medical practice resulting from their global pediatrics training, we identified six categories describing areas of impact: 1) global health knowledge and tools, 2) global citizenship, 3) clinical practice, 4) humanism, ^15^ 5) career development, and 6) valued relationships. Because we identified the same categories within the responses to both open-ended questions, we have presented the categories with examples of specific impacts coded to each category from both open-ended questions and the frequency of each category in a single table (Table 3).

**Table 3:** Categories of written comments regarding additional impacts of global pediatrics training, with examples and the frequency of each category (29 comments)

Category	Examples of impacts or influences on respondents’ practice of medicine coded to each category	No. (%) of comments that contained the category
Global health knowledge and tools	Increased global health medical knowledge Increased understanding of health disparities Increased understanding of social determinants of health	14 (48)
Global citizenship	Broadened vision for how physicians can affect underserved populations Broadened perspective to think globally Recognized the importance of global partnerships in global health	10 (34)
Clinical practice	Increased comfort in refugee screening Increased confidence with travel medicine Increased confidence caring for immigrants and refugees	9 (31)
Humanism*	Increased empathy for patients Increased cultural competence Acquired skills to work in an ethically responsible way	9 (31)
Career development	Impacted subspecialty choice Increased interest in research Increased commitment to caring for immigrant and refugee communities	6 (21)
Meaningful relationships	Gained career mentors Networked with others interested in global health Provided community during residency	4 (14)

* Based on the Arnold P Gold Foundation definition of humanism in health care, which is “characterized by a respectful and compassionate relationship between physicians, as well as all other members of the healthcare team, and their patients. It reflects attitudes and behaviors that are sensitive to the values and the cultural and ethnic backgrounds of others.” ^15^

### Use of procedural skills while working abroad.

Of the 57 respondents to this question, 41 (72%) reported participating in some form of procedural training for low-resource settings. Eighteen respondents reported using these skills while abroad. Table 4 summarizes these findings. The skills used most frequently were neonatal resuscitation, bubble continuous positive airway pressure, a water bottle spacer for metered dose inhaler administration, oxygen delivery devices, and bag–valve mask ventilation.

**Table 4:** Frequency of use of procedural skill/device in resource-limited settings

Device or procedure name	Frequency		
	1–5	6–10	> 10
Neonatal resuscitation	10	0	3
Bubble CPAP	7	2	2
Spacer for MDI	7	0	2
Oxygen delivery devices	4	2	4
Bag–valve mask ventilation	6	0	4
IO needle placement	3	1	1
Exchange blood transfusion	4	0	0
IV fluids without pumps	0	0	3
Burn treatment/dressings	1	1	1
Pleur-evac chest tube drainage	1	1	0
Chest simulation model	0	0	0

CPAP = continuous positive airway pressure; IO = intraosseous; IV = intravenous; MDI = metered dose inhaler.

## DISCUSSION

This study suggests that global pediatrics track graduates perceive that their global pediatrics training affected their career development, clinical skills, use of adapted procedural skills, as well as their personal development and global perspective. We found that most respondents (84%) planned to work in global health both before and after participating in the track, with 69% reporting they were engaged in global health work at the time of the survey. The global pediatrics track graduates comprise 28% of the total number of eligible graduates from the institution’s pediatric and medicine–pediatric residency programs. The respondents reporting current engagement in global health work therefore represent 8.6% of eligible residents. These percentages compare favorably with two recent American Academy of Pediatrics surveys. In a 2016 survey of graduating pediatric residents, 42% reported career plans to participate in global health domestically or internationally. ^12^ In the second, a survey of practicing pediatricians in 2017, 32% reported plans to work in a low- or middle-income country and 5.1% reported overseas global health experiences in the previous 12 months. ^16^

Respondents’ interest in working in global health remained largely constant from before to after track participation, likely reflecting their self-selection into a global health track, similar to the conclusion of the study by Gupta et al. ^11^ of Yale internal medicine residents. However, respondents reported that their specific interests were influenced by participation in the track, including sub-specialty choice, increased interest in global research and public health, as well as working with immigrant and refugee populations. This highlights the need for global pediatrics training programs to continue developing robust curricula in these areas, including training in appropriate research content, ethics, and methods, with an emphasis on building equitable research collaborations with international partners.

Respondents also indicated the importance of meaningful relationships established through their participation in the global pediatrics track, including valued mentorship relationships and career networking. This underscores a need for global pediatrics training programs to have a strong network of mentors available to their trainees, with wide-ranging expertise and interests to support the varied and developing career paths of their trainees. Indeed, the respondents who reported they were engaged in global health work at the time of the survey demonstrate a wide range of interests in global health. Respondents represented current work in 17 different countries as well as engagement with immigrant and Native American communities in the United States. Importantly, they described working in all areas of global health: clinical, education, research, and advocacy.

In addition, respondents perceived that global pediatrics training enhanced their clinical skills significantly, including their ability to elicit information about cultures and beliefs from patients and their families, provide cost-conscious care, and perform physical examinations skillfully. This finding is consistent with previous studies. ^9^^,^ ^17^^,^ ^18^ Respondents also noted that global pediatrics training increased their confidence in specific skills such as refugee screenings and travel medicine. As individuals and populations are increasingly mobile, there is a growing need for U.S.-based pediatricians to be well trained in the care of children traveling to and from the United States. ^19^ Global pediatrics tracks play a key role in preparing pediatricians to provide skilled, holistic care to all children.

Global pediatrics track participants describe increases in empathy for patients, and an enhanced ability to work in an ethically responsible way. Of note, across all levels of training, empathy is shown to decrease over the course of medical training. ^20^ Our study supports the findings from Lauden et al., ^21^ who showed that compared with non-global health track participants, global health track participants have greater levels of empathy. The incorporation of global health education into trainee curricula has the potential to address issues surrounding empathy erosion, although further research is required in this area.

Although it is well established that global pediatrics training results in increases in medical knowledge, including physical examination skills, ^22^^,^ ^23^ our study further highlights the broadening of trainee perspectives of the underlying drivers of morbidity and mortality. Global pediatrics track participants describe enhanced understanding of how social determinants of health affect underserved populations and how physicians may affect underserved populations positively. This widening of perspective suggests that global pediatrics training can encourage global citizenship, including the recognition that pediatricians can contribute to health for all children, and can have an impact beyond their local communities through service and advocacy.

To our knowledge, our study is the first to quantify the use of adapted procedural skills taught to trainees prior to their international electives. Several articles have been published that have proposed guidelines for training medical providers on how to substitute high-resource equipment for low-cost, high-access equipment in acute medical situations ^5^^,^ ^24^; however, no study has been published to evaluate the use of these guidelines. In this survey, the high use of neonatal resuscitation skills, bag–mask ventilation, and oxygen delivery devices is relatively unsurprising as these categories encompass a range of skills that can be used with equipment that is found readily in low-resource settings. It is notable that improvised bubble continuous positive airway pressure and metered dose inhaler spacer construction were used at a relatively high frequency, which may be reflective of the simplicity and ease of their construction as well as a high demand for these devices in low-resource settings. In contrast, the chest tube drainage system, a more complicated skill, was used less widely by our cohort. Last, the management of intravenous fluids without pumps was not often used by our residents. This could be reflective of the hosts’ resources, such as medical students, residents, and nurses knowledgeable in calculating and delivering the appropriate volume of intravenous fluids.

Our study has several limitations. First, we did not define global health work explicitly to mean work in resource-constrained communities in the United States and abroad in the survey. Respondents may have defined global heath differently, ^25^ which could have led them to underreport what we or others may consider global health work. Second, recall bias and selection bias are inherent in survey data. It is possible that individuals who were engaged in global health work were more likely to respond than those who were not at the time of the survey. Third, the survey was sent during the first wave of the coronavirus disease 2019 pandemic, perhaps limiting participation. Our data may underrepresent the actual number of our graduates involved in global pediatrics activities and careers. With a 62% response rate, track graduates who are working in high-volume global health settings may not have had the opportunity or Internet access to respond to an e-mail-based survey. Nonetheless, the number of respondents is comparable to similar studies evaluating global health education in pediatric training programs. ^10^^–^ ^12^ In addition, responses may reflect uncertainty regarding international travel in light of the pandemic. Fourth, our data only reflect a subset of physicians who obtained a global pediatrics track certificate from a single institution, which may limit the generalizability of our findings. Last, because procedural skills use was not correlated with specific electives or career paths, we were unable to account for those graduates who have been or are involved in activities in which adapted procedural skills are not needed (i.e., advocacy, research).

Future work on the long-term outcomes and impacts of global pediatrics training should include larger studies with more participants, including international residents hosted by U.S. academic centers, multiple global pediatrics tracks, and global partners. These larger studies are needed to understand more completely the range of impacts of global child health education programs as well as program modifications needed to increase the competency of the global child health workforce. In addition, given the small number of respondents who indicated they did not plan to work in global health before participating in the global health track, further research is needed to understand more fully the motivations for global health track participation outside of longer term career interests. Further investigation of the procedural skills necessary to know before working in low-resource areas, regardless of whether participants are in the United States or abroad, as well as revisions and modifications to skills training are needed. Last, our survey tool could be refined to elucidate more details regarding global pediatrics track graduates’ current global pediatric work.

Our study adds to the growing body of knowledge about the multifaceted impacts of global child health education on trainees and, by extension, on global health systems. Our research suggests that graduates of a global pediatrics track during residency perceive a long-lasting impact, up to 12 years after training, in the critical areas of career development, commitment to global health work and underserved populations, clinical skills, and global citizenship. Therefore, we believe that resident training in global pediatrics has the potential to improve health care across cultures and ethnicities, promoting health equity both locally and globally.

## Supplemental Material


Supplemental materials
