# Supplementary material for: Long-Term Impact of Global Pediatrics Curriculum, Experience, and Mentorship in Pediatric Residency
Source: Am J Trop Med Hyg. 2022 Feb 7;106(4):1057–62. doi: 10.4269/ajtmh.21-1014 (PMC8991360; doi:10.4269/ajtmh.21-1014)
Supplement: Supplementary file 1 [file tpmd211014.SD1.pdf]

Appendix 1: Survey Instrument

1. Has the global pediatrics training you received in residency impacted your career choices? (Yes or No)
2. If yes: Please indicate how global pediatrics training affected your plans in each of the following areas prior to and after participation in the track: (PRIOR to participating in the global pediatrics track: yes/no and After participating in the global pediatrics track: yes/no
    - Were you planning to work in global health?
    - Were you planning to work with underserved populations globally?
    - Were you planning to work with domestic underserved populations?
    - Were you planning to incorporate public health into your clinical work?
    - Were you planning to include research as part of your career?
    - Were you planning to pursue subspecialty training?
3. Describe any other impact of global pediatrics training.
4. Did the global pediatrics training improve your skills in the following areas of clinical practice? (Yes, no, or unsure)
    - Physical examination
    - Cost-conscious care (e.g. choice of labs/imaging, medications and /or use of medical supplies)
    - Recognition of "sick" versus "not sick"
    - Eliciting information about cultural beliefs and practices which impact my patient's health Understanding of medical terminology in languages other than English
5. Please provide more information about any of the skills above or comment on how the global pediatrics training has influenced your practice of medicine.
6. Are you still engaging in global health activities either in the United States or abroad? (Yes or No)
7. If yes to Question 6: What do you do? (Please include country, type of work done abroad and ongoing partnerships or research).
8. Did you receive Procedural Education for Adaption to Resource Limited Settings (PEARLS) developed by the University of Minnesota Global Pediatrics division? (Yes or No)
9. If no to Question 8: Were you taught simple procedures or procedural adaptations to prepare for work in resource-limited settings? (Yes or No)
10. If yes to Question 8 or 9: Did you utilize any of the procedural skills while working abroad? (Yes or No)*
11. If yes to Question 10: State country(ies) where procedure skills were utilized
12. If yes to Question 10: What procedural skills did you use?
    - Bubble CPAP
    - Intraosseous Needle Placement
    - Exchange Blood Transfusion
    - IV fluid delivery without infusion pumps
    - Spacer for MDI
    - Bag Valve Mask Ventilation (BVM)
    - Neonatal Resuscitation
    - Oxygen Delivery Devices
    - Chest Simulation Model
    - Pleurovac Chest Tube Drainage
    - Burn Treatment/Dressings
    - Other (please enter procedure below)
13. If any options from Question 12 were selected: How many times?
    List selected answers from question 12.with response options:
    - 1-5
    - 6-10
    - >10
    - Unsure about # of times

End of Survey

*If no to Question 10: End of Survey
